# Statistical Mechanics Metrics in Pairing and Parsing In Silico and Phenotypic Data of a Novel Genetic NFκB1 (c.T638A) Variant

**DOI:** 10.3390/genes14101855

**Published:** 2023-09-24

**Authors:** Eman N. Chaudhri, Jessica M. Abbott, Naeyma N. Islam, Caleb A. Weber, Mathew A. Coban, Ahmet Bilgili, Jacqueline D. Squire, Sarah Mantia, Klaas J. Wierenga, Thomas R. Caulfield

**Affiliations:** 1 Department of Neuroscience, Mayo Clinic, Jacksonville, FL 32224, USA; echaudhri@alfaisal.edu (E.N.C.); abbott.jessica2@mayo.edu (J.M.A.); islam.naeyma@mayo.edu (N.N.I.); weber.caleb@mayo.edu (C.A.W.); ahmet.bilgili@ufl.edu (A.B.); 2 College of Medicine, Alfaisal University, Riyadh 11533, Saudi Arabia; 3 Department of Cancer Biology, Mayo Clinic, Jacksonville, FL 32224, USA; coban.mathew@mayo.edu; 4 Department of Allergy-Immunology, Mayo Clinic, Jacksonville, FL 32224, USA; squire.jacqueline@mayo.edu; 5 Department of Clinical Genomics, Mayo Clinic, Jacksonville, FL 32224, USAwierenga.klaas@mayo.edu (K.J.W.)

**Keywords:** NFκB1 variant, NFκB1 mutation, molecular modeling, molecular dynamics, common variable immunodeficiency, autosomal dominant

## Abstract

(1) Background: Mutations in *NFκB1*, a transcriptional regulator of immunomodulating proteins, are a known cause of inborn errors of immunity. Our proband is a 22-year-old male with a diagnosis of common variable immunodeficiency (CVID), cytopenias with massive splenomegaly, and nodular regenerative hyperplasia of the liver. Genetic studies identified a novel, single-point mutation variant in *NFκB1*, c. T638A p. V213E. (2) Methods: Next-generation panel sequencing of the patient uncovered a novel single-point mutation in the *NFκB1* gene that was modeled using the I-TASSER homology-modeling software, and molecular dynamics were assessed using the YASARA2 software (version 20.14.24). (3) Results: This variant replaces valine with glutamic acid at position 213 in the NFκB1 sequence. Molecular modeling and molecular dynamic studies showed altered dynamics in and around the rel homology domain, ankyrin regions, and death domain of the protein. We postulate that these changes alter overall protein function. (4) Conclusions: This case suggests the pathogenicity of a novel variant using protein-modeling techniques and molecular dynamic simulations.

## 1. Introduction

Nuclear Factor kappa-light-chain-enhancer of activated B cells 1 (NFκB1) is a transcription factor protein encoded by the *NFκB1* gene located on chromosome 4q24. It is one of five members of the NFκB family of transcription-regulating proteins, also including NFκB2, RelA, RelB, and c-Rel. These proteins form numerous homo- or heterodimer complexes with each other to initiate a variety of downstream signaling pathways involved in immunity [1,2]. In the more widely understood canonical NFκB pathway, NFκB1 binds to RelA to form an NFκB complex. This complex is normally inhibited by IkB proteins that sequester the inactive NFκB complex in the cytoplasm. When IKK proteins phosphorylate IkB, tagging it for ubiquitination and degradation by the 26 S proteasome, IkB is released from the NFκB complex and the NFκB complex is then free to move into the nucleus and begin transcription of target genes, mainly including proinflammatory elements [3,4]. NFκB1 is expressed in different types of cells that secrete cytokines, chemokines, and other chemical factors and mediators of inflammation and healing. It is the most highly expressed transcription factor in macrophages, and, interestingly, its knockout has been shown to increase the pro-inflammatory activity of these cells [5]. The inappropriate activation of NFκB proteins can lead to a variety of autoimmune conditions, including arthritis, lung fibrosis, asthma, and glomerulonephritis. Furthermore, the over-inhibition of NFκB proteins may cause delayed immune response, cellular apoptosis, and inappropriate immune cell development [6,7].

**Figure 1:**
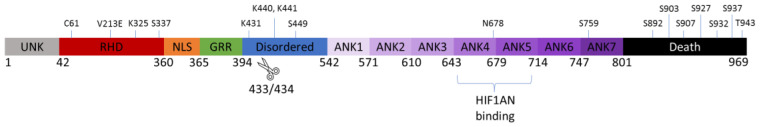
A 2D domain map of NFκB1 protein labeled with numbered amino acid positions. Both the V213E mutation and common NFκB1 post-translational modifications are labeled (key: **UNK**: Unknown region; **RHD**: Rel Homology Domain; **NLS**: Nuclear Localization Sequence; **GRR**: Glycine-Rich Region; **ANK** = Ankyrin; **Scissors Graphic:** Location of processing point from p105 to p50).

The previously described 2.3 Å resolution crystal structure of the NFκB1 p50 homodimer bound to a palindromic κB site (PDB 1NFK) is comparable to other members of the immunoglobulin superfamily in that the Rel homology domain folds into two regions [8]. NFκB proteins recognize and bind consensus DNA elements called κB sequence sites. NFκB1 is a protein consisting of 969 amino acids [9]. It is divided into separate domains, as depicted in color in Figure 1. The *NFκB1* gene encodes the p105 precursor, which is most often co-translationally processed into the p50 subunit (433 amino acids) that can bind DNA [10]. Translation of NFκB1 mRNA begins at the N-terminus, with the first functional domain being the rel homology domain (RHD). The RHD is responsible for DNA recognition and interaction with other NFκB proteins [11]. This domain is followed by the nuclear localization sequence (NLS), which mediates NFκB1’s entrance into the nucleus from the cytoplasm, and then the glycine-rich region (GRR), which functions as a processing signal for the generation of the p50 subunit [12]. The GRR is then followed by multiple copies of ankyrin (ANK) repeats, which stabilize the NFκB1 p105 protein and inhibit it from binding to DNA [13]. After the last ankyrin region is the death domain, which is responsible for the signal-induced proteolytic cleavage of NFκB1 p105 into p50 [14,15].

NFκB1 has been implicated in crucial cellular processes such as cell survival, proliferation, inflammation, and the adaptive immune response [16]. *NFκB1* mutations known to cause p50 haploinsufficiency have been associated with Common Variable Immunodeficiency (CVID), autoinflammatory and rheumatologic diseases, gastrointestinal manifestations, lymphoproliferation, and an increased risk of many types of opportunistic infections ranging from viral to fungal in nature [17]. One study even identified loss-of-function variants in *NFκB1* as the most common monogenic cause of CVID in Europeans [18]. CVID is a heterogenous disorder characterized by hypogammaglobulinemia, impaired immunoglobulin production, and susceptibility to infections [19]. In addition to increased susceptibility to infection, especially of the sinopulmonary tract, other noninfectious autoimmune and inflammatory conditions commonly appear in CVID cases. Manifestations may include thrombocytopenia, neutropenia, splenomegaly, or even lymphoma [20]. CVID is a rare inborn error in immunity diagnosed in an average of 0.676 per 100,000 individuals globally [21]. CVID affects individuals beginning in early adulthood and expresses variable phenotypes in patients. In a study conducted by Lorenzini et al. in 2020, 157 individuals displayed variation in their CVID phenotype caused by an *NFκB1* variant, presenting effects including autoinflammation, autoimmunity, lymphoproliferation, enteropathy, and a variety of infections [16]. Common types of mutations involved include predicted loss-of-function and loss-of-expression mutations [22].

In this report, we present a patient with a novel point mutation T638A in the sequence of *NFκB1* (NM_003998.3). This mutation substitutes valine for glutamic acid at residue 213 (V213E), likely causing pathogenicity and leading to a clinical presentation similar or equivalent to that of CVID. We support this theory with protein molecular modeling techniques and molecular dynamic simulations. Additionally, we discuss the patient’s unique variant in *NFκB1*, comparing his symptoms and family history to those of existing cases.

## 2. Case Report

The proband is a 22-year-old male who was first assessed via Telemedicine due to COVID-19 restrictions. This patient is the only child to a mother who has a history of anxiety, migraines, and seizures and a father for whom little clinical history is available. Consanguinity within the family was denied.

In his early medical history, the proband reported suffering frequent illnesses as a toddler. An eventual diagnosis of asthma and allergies was made. The patient did not benefit from treatment, and further medical evaluation revealed common variable immunodeficiency (CVID). The patient received intravenous immunoglobulin (IVIG) and has been on IVIG therapy since. While there has been a decrease in the frequency of infections, our proband still experiences pneumonias, sinusitis, and bronchitis episodes. He was well until the age of 19, at which time a massively enlarged spleen was identified upon physical examination. He received a splenectomy, and his spleen weighed around 7.5 lb when removed. The patient developed right upper quadrant pain and underwent a cholecystectomy that identified biliary sludge and stones and transaminitis with elevated alkaline phosphatase levels.

The patient had undergone genetic testing a couple of years prior to this examination, including using an Invitae primary immunodeficiency gene panel, which yielded negative results. The proband agreed to more intensive multi-gene panel testing for primary immunodeficiencies. The second primary immunodeficiency gene panel run led to the discovery of a point mutation in *NFκB1*. To the best of our knowledge, this V213E (Val213Glu) variant has not been reported elsewhere; thus, it can be deemed a novel variant and one of uncertain significance (VUS). In the time since the proband’s genetic testing, he has continued to follow up with the Department of Clinical Genomics as well as the Allergy and Immunology Department. Through out-patient care, the proband is currently on anti-bacterial, anti-fungal, and analgesic medications along with necessary inhalers.

## 3. Materials and Methods

### 3.1. Ethical Compliance

Standard evidence-based medical care was followed in treating the patient in this case, and all procedures followed were in accordance with the ethical standards of the responsible committee on human experimentation (institutional and national) and with the Helsinki Declaration of 1975, as revised in 2000 [23]. Written informed consent for genetic analysis and all other testing was obtained from the patient.

### 3.2. Molecular Modeling

The sequence of human Nuclear Factor Kappa-light-chain-enhancer of activated B cells Subunit 1 (known as NFκB1), a protein encoded by the *NFκB1* gene on Chromosome 4 (4q24), was taken from the NCBI Reference Accession Sequence NP_003989.2, which encodes the amino acid sequence included in Appendix A Table A1 and was used for computer-assisted modeling. Multiple model-building algorithms were utilized to construct a full-length 3D model of NFΚB1; these results were compared, and the best model was determined to be the result of the I-TASSER algorithm [24]. Alphafold was examined for comparison where appropriate [25]. Our computational platform for in silico variant analyses has been extensively explored in recent years [26,27,28,29,30,31,32,33,34,35].

### 3.3. Molecular Dynamics Simulations

Since proteins are not static, we employed molecular dynamics simulations to investigate the impact of the novel mutation V213E on the NFκB1 protein’s intracellular/intranuclear dynamics. The use of simulations and other techniques for pathogenicity determination of missense variants has been demonstrated to be successful in making these “calls” [36,37,38,39]. We conducted all-atom unbiased molecular dynamics simulations (MDS) on the WT and V213E NFκB1 models using YASARA with the YASARA2 force field [40], as previously described in work conducted in our lab [38]. These simulations were performed to investigate the impact of the proband’s variant on the conformational dynamics of NFκB1 and to suggest the phenotypic significance of the variant (i.e., whether it was pathogenic or not). Briefly, the NFκB1 models were subjected to energy minimization with relaxed restraints using the steepest-descent Polak–Ribiere conjugate gradient method [41]. The simulation box was constructed 15 Å from the nearest protein atom; subsequently, the box was filled with TIP3P waters at a density of 0.997 g/L with Na+/Cl− at 150 mM, temperature set to 310 K, pressure at 1 bar, and pH of 7.4. Long-range Coulombic forces were calculated utilizing particle mesh Ewald with a 7.86 Å cutoff for periodic boundary conditions [42]. Simulations were carried out for 100 ns on each system. The program Visual Molecular Dynamics (VMD) [43] was used for analyses of the simulations and trajectory analyses [44]. Images were generated using the program PyMOL [45]. 

## 4. Results

### 4.1. Gross Structure and Domain Map of Wild Type and Novel Variant V213E NFκB1

**Figure 2:**
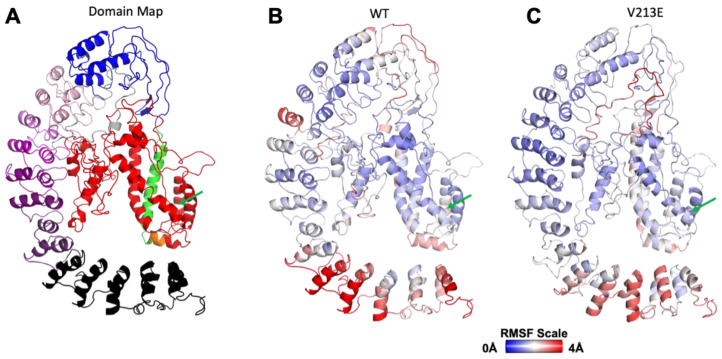
Three-dimensional domain maps of NFκB1 molecule and RMSF color maps of WT and V213E NFκB1 molecules created using I-TASSER software(version 2023): (**A**) 3D domain map depicted within I-TASSER-predicted gross NFκB1 structure labeled according to 2D color domain map in Figure 1; (**B**) wild-type gross NFκB1 structure colored via RMSF; (**C**) mutated V213E NFκB1 gross structure colored via RMSF (key: **I-TASSER:** Iterative Threading Assembly Refinement; **WT**: Wild Type; **RMSF**: atomic Root Mean Square Fluctuation scale (in angstroms); **Green Arrow:** amino acid position 213).

The results of our proband’s genetic panel testing showed a novel mutation in position 638 of the NFκB1 gene-coding sequence, corresponding to amino acid position 213 in the protein. After creating our full-length homology model, we were able to determine the function and location of each domain of the NFκB1 molecule, as shown in Figure 1 and Figure 2A–C. 

As shown in Figure 1, our novel mutation V213E falls in the Rel Homology Domain (RHD) depicted in red, from positions 42–360. This domain is arguably the most functionally important as it is responsible for proper DNA recognition. There was no gross change in structure in the RHD from the WT to the novel mutant versions of our modeled NFκB1. Both structures were similar macroscopically and microscopically. 

### 4.2. Molecular Dynamic Simulations via YASARA2 Software

**Figure 3:**
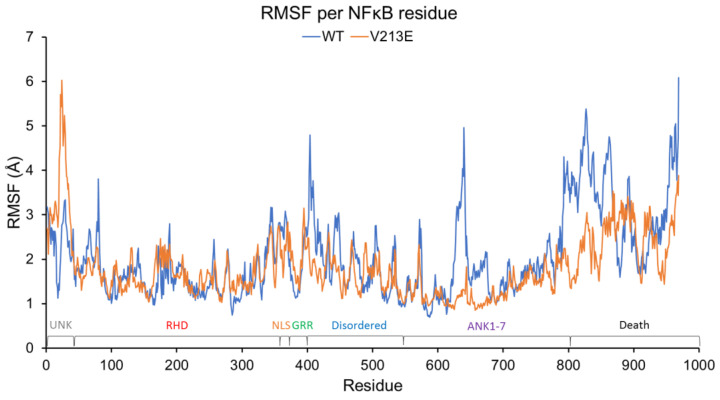
Graph depicting the RMSF in angstroms per residue of WT and V213E mutant NFκB1 molecule in YASARA2-software-conducted molecular dynamic simulation. Domains are mapped and color-labeled according to Figure 1 and Figure 2 (key: **WT**: Wild Type; **RMSF**: Root Mean Square Fluctuation).

**Figure 4:**
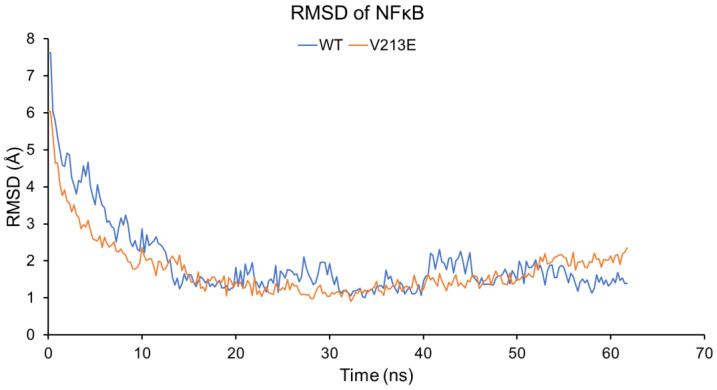
Graph depicting the RMSD in angstroms over time in YASARA2-software-conducted molecular dynamic simulation of WT and V213E NFκB1 protein (key: **WT**: Wild Type; **RMSD**: Root Mean Square Deviation).

The deviation of motion, as depicted in Figure 3 and Figure 4, might indicate an aberrant ability to be cleaved or post-translationally modified (PTM) or to interact with important partners such as DNA or other proteins at nearby residues.

Figure 3 and Figure 4 display important fluctuation and deviation differences between the amino acids that make up WT and V213E NFκB1 in various regions of the protein. Although there is no significant difference in RMSF at position 213 between the variant and WT NFκB1, a notable feature that can be clearly seen in Figure 3 is the heightened fluctuation of amino acid location in residues 10–40 of V213E NFκB1 compared to the WT. Interestingly, the amino acids of this region have not been characterized in the literature into an ordered functional domain. However, these amino acids lie adjacent to arguably the most important domain of NFκB1: the RHD. The significantly increased mobility of these amino acids may cause physical or electrostatic interference and blockage of RHD’s binding to DNA. Furthermore, one can see a notable decrease in the dynamics of the RHD surrounding amino acid 80 in the variant. This dynamic difference in the RHD may have a direct impact on the protein’s main function of recognizing target genes on the DNA for transcription. Furthermore, Figure 3’s depiction of the RMSF of each amino acid residue shows a lack of fluctuation of the residues in V213E NFκB1 from roughly position 600 to 969 (the C-terminus) compared to the WT, for which abundant fluctuation is depicted. This area corresponds to the last ankyrin-binding regions and whole death domain of the NFκB1 molecule, which are typically cleaved during the processing of NFκB1. These suppressed dynamics in the variant can specifically be seen in the region between residues 793 and 866, involving the Ankyrin 7 (ANK7) and the death domain. This is significant in that the variant may affect the protein’s proteolytic cleavage ability in its p105 precursor form, leading to both a lack of functional p50 and a build-up of p105. Interestingly, p105 is currently being studied as an inhibitor of the NFκB pathway in a similar manor to IkB because there is major structural homology between the ANK regions of p105 and the IkB isoforms [1]. The differential dynamics in this region may also interfere with binding to various partners and overall protein stability. Specifically, the region between residues 623 and 676 that varied dynamically in WT NFκB1 may influence the protein’s binding with partners like Hypoxia-Inducible Factor 1-α Inhibitor (HIF1AN) [46]. There is also notably more fluctuation in positions 403–431 of the variant NFkB1 corresponding to the already-disordered domain.

Figure 4 depicts the deviation in the atomic position of the overall protein rather than the individual residues of the protein as analyzed in Figure 3. In addition to these regional/per-residue views, there is a notable, though minor, impact on global dynamics. In Figure 4, the RMSD is similar in both the WT and V213E variant mutant forms of NFκB1; however, there is slightly less deviation in the V213E variant mutant form, indicating a possible increase in large-scale conformational stability. This result strengthens our prediction of increased p105 stability and decreased readiness to be cleaved into the p50 form as a result of the V213E variant. This stability increase could also have impacts on the protein’s ability to interact with other binding factors and DNA, thereby altering its overall function. Due to the variation in the dynamics of the mutant molecule in comparison to the WT NFκB1, aberrant activity is likely indicative of a partial or complete loss of function or potentially a toxic gain of function. Based on available data gathered through homology modeling and molecular dynamic simulation testing, the V213E mutation represents a detriment in that it may result in abnormal NFκB pathway signaling, leading to an altered response to cell stress and immune function.

## 5. Discussion

The current American College of Medical Genetics (ACMG) standards classify this variant as one of uncertain significance given that it falls under the following criteria: (1) it is located in a well-established functional domain (PM1), (2) it is absent in controls in the Exome Sequencing Project or the 1000 Genomes Project (PM2), (3) it has computational evidence suggests that has a deleterious effect (PP3), and (4) it is a missense variant in a gene for which primarily truncating variants are known to cause diseases (BP1) [47]. VUSs are a hurdle that researchers and clinicians alike are trying to overcome. With the advent and abundant use of next-generation sequencing, an increasingly large number of variants are being discovered that have little to no clinical or functional validation data with which to establish whether the variant is pathogenic or benign. The computational modeling tools we used here are employed to add a layer of evidence to current pathogenicity predictions and give increased confidence in the effects of unique variants [48].

With numerous mutations that could be implicated in immunodeficiencies of unknown origin, it is difficult to tell which variants are pathogenic and to what extent. Not all mutations contribute to the pathogenicity of a disease process or cause the dysregulation of crucial processes in the body. However, as supported by this case and other instances of CVID, a patient’s clinical presentation is generally a strong indicator of the potential pathogenicity of a novel mutation. While the homology models do not show gross differences or abnormalities of the variant compared to the WT structures of NFκB1, our MDS results have shown dynamic variance in areas of the NFκB1 protein chain that themselves do not contain the V213E mutation. For this reason, we emphasize the value and importance of our novel dynamic simulation approach as opposed to traditional methods of evaluating only static structure predictions. What this reveals is a play of events downstream of the mutation that affects its intracellular dynamics and interaction with DNA, binding factors, and proteolysis factors. It is already well established that a dysregulation of the NFκB pathway of any type can result in chronic inflammation, immunodeficiencies, and cancers. Moreover, mutations in NFκB pathway components have been implicated numerous times in the development of inflammatory conditions and immune dysregulation [49].

Based on the dynamic variations in the novel V213E NFκB1, we can say that this mutation plausibly interferes with the normal function of the protein. The dynamic variance of the death domain of the V213E NFκB1 protein indicates a further need to determine the exact change in the molecular function of the protein for future drug discovery and the recognition of the same family of diseases. A loss of proteolytic cleavage ability could result in p105 buildup that may have the gained function of inhibiting the NFκB pathway. Alternatively, the differential dynamics in the RHD region may directly alter its ability to bind its target DNA. Altered dynamics leading to instability in and around the RHD region may obstruct the necessary DNA-binding conformation of NFκB1 in the variant. However, we cannot identify with certainty the mechanism by which this deviation may influence DNA binding. Also, not every translated NFκB1 protein will behave in the same way, so multiple dynamic effects are likely at play due to this novel mutation. Regardless of the exact effect on its molecular pathway, it can be concluded that the adjusted protein dynamics visualized through our computational tools may lead to altered protein interaction capabilities that are indicative of potential pathogenicity.

We have put forth our interpretations of DNA-binding affinity because affinity is composed of an on rate, where the unbound components meet and associate, as well as an off rate, where the bound components separate. Since DNA binding would be predicated on the RHD adopting a specific subset of DNA-binding competent conformations, and as the RHD and its surrounding regions have conformational variability in V213E, it is less likely to adopt one of those conformations and is expected to have a slower on rate for DNA binding.

To further corroborate these findings, REVEL, a combined score of 13 modern in silico pathogenicity prediction programs that accounts for various biochemical alterations in variants, predicted that the V213E NFκB1 variant is pathogenic. On a scale of 0–1, where the magnitude increases with the likelihood of pathogenicity, this variant was scored as 0.879. A critical interpretation of the pathogenicity cutoff would place a pathogenic variant at 0.75 or higher; this variant far surpasses that mark [50]. 

We would like to emphasize that our presented in silico analyses may only be considered one unit of evidence in the complex undertaking of comprehensive variant analysis. Our interpretation of pathogenicity is a prediction based solely upon our novel collected data. Our results and theoretical downstream effects should not be taken as a definitive judgement on pathogenicity or mechanism of action. Further experimental and clinical evidence will be needed to solidify this determination. Here, we offer a novel computational approach to the analysis and visualization of NFκB1 V213E with the intent to provide supporting evidence towards a pathogenicity judgement and stimulate interest in further research efforts surrounding this variant. 

Molecular modeling and other in silico analyses have made considerable strides in the field of variant analysis in recent years. For instance, in a study published in 2023, a novel missense variant in *CYB5R3* was found in a family associated with recessive congenital methemoglobinemia. Molecular dynamic simulations and other in silico tools were used to approach the analysis of the variant of interest and revealed a likely pathogenic dynamic prediction [51]. In another case, clinical assessment accompanied by molecular dynamics simulations of two cases of novel missense variants in the transcription factor-encoding gene *NR2F1* for Bosch–Boonstra–Schaaf optic atrophy syndrome were indicative of variant pathogenicity [52]. In yet another instance, both in silico and in vitro functional analyses of a *TGFBR2* VUS with a clinical diagnosis of Marfan syndrome confirmed the pathology of the variant and suggested the diagnosis of a very similar syndrome, Loeys–Dietz syndrome [52]. Molecular modeling has been shown to be of use in analyzing single variants alone [53], multiple variants in one gene [52,54,55,56,57,58], and multiple genes in one disease [59]. Interestingly, molecular modeling has also been of use in visualizing the interaction between different variants of SARS-CoV2 and the human ACE2 receptor [60,61]. We share these studies to highlight the growing importance of in silico contributions to the field of genetic interpretation [62]. Moving a step beyond variant analysis alone, examples from continued in silico technique development that enriches the ability to make useful inferences from gene variation and resulting protein states have been broadly fruitful for determining the druggability of disease targets of interest. In view of this, there is hope that *NFκB1* shall benefit [63,64,65]. 

Our simulation results and interpretations also yield testable hypotheses for laboratory-based research. Ideally, we would perform these experimental approaches to show that V213E restricts the processing of p105 into p50, hinders the ability of p50 to bind DNA, and reduces the expression of NFκB1 transcriptional targets. Protein-binding assays such as microscale thermophoresis for assessing WT vs. V213E NFκB1 (in both p50 and p105 forms) affinity for RelA, or DNA-binding assays such as electrophoretic mobility shift assays to assess the affinity of NFκB1 WT vs. V213E for their relevant target DNA sequences, may be indicative of functional impairment. Binding assays with HF1AN or other binding partners could confirm the prediction that this variant interferes with protein interactions outside of the canonical NFκB pathway. Time course digestion assays could confirm aberrant cleavage susceptibility for processing into mature NFκB1 (p50). These protein–protein and protein–DNA interactions may also be visualized using intensive computational methods. Cell-based assays could offer the next level of evidence in variant analysis. The most-definitive analyses would examine relevant functional events in WT vs. V213E NFκB1 human immune cells, either collected from the patient through peripheral blood mononuclear cell collection or using an available cell line. The methods of our analyses pave the way for the evaluation of more *NFκB1* mutations and possibly the categorization of *NFκB1* mutations into the variety of immunodeficiencies, inflammatory diseases, and cancers that they cause. 

Although knowledge regarding the phenotype and expressivity of the V213E mutation is limited due to its recent discovery and manifestation in only one patient, it serves as a foundation for updating mutation databases that encompass gene mutations and pathogenic variants. This variant has yet to be seen in ProteinPaint or HGMD databases of clinically relevant variants [66,67]. It is not accounted for in GnomAD either, a database containing a globally diverse set of genome-sequencing results [68]. An update of databases containing *NFκB1* variants and correlating phenotypic expression should be made to better discern how various mutations manifest clinically. Patients with unexplained immunodeficiency-like symptoms and presentation need to continue to be considered for genetic testing to determine the most likely culprits of their phenotypic presentations. This will be critical in determining clinical prognoses, personalized medical treatment approaches, and further preclinical molecular studies and drug design. 

## Data Availability

Modeling and simulation data will be made available upon request to the corresponding author.

## Appendix A

**Table A1:** Next-generation sequence of proband’s NFκB1 gene with a single-point mutation resulting in substitution of valine with glutamic Acid at position 213.

“MAEDDPYLGRPEQMFHLDPSLTHTIFNPEVFQPQMALPTADGPYLQILEQPKQRGFRFRYVCE GPSHGGLPGASSEKNKKSYPQVKICNYVGPAKVIVQLVTNGKNIHLHAHSLVGKHCEDGICTVT AGPKDMVVGFANLGILHVTKKKVFETLEARMTEACIRGYNPGLLVHPDLAYLQAEGGGDRQL GDREKELIRQAALQQTKEMDLSVERLMFTAFLPDSTGSFTRRLEPVVSDAIYDSKAPNASNLKIV RMDRTAGCVTGGEEIYLLCDKVQKDDIQIRFYEEEENGGVWEGFGDFSPTDVHRQFAIVFKTPK YKDINITKPASVFVQLRRKSDLETSEPKPFLYYPEIKDKEEVQRKRQKLMPNFSDSFGGGSGAGAG GGGMFGSGGGGGGTGSTGPGYSFPHYGFPTYGGITFHPGTTKSNAGMKHGTMDTESKKDPEG CDKSDDKNTVNLFGKVIETTEQDQEPSEATVGNGEVTLTYATGTKEESAGVQDNLFLEKAMQL AKRHANALFDYAVTGDVKMLLAVQRHLTAVQDENGDSVLHLAIIHLHSQLVRDLLEVTSGLISD DIINMRNDLYQTPLHLAVITKQEDVVEDLLRAGADLSLLDRLGNSVLHLAAKEGHDKVLSILLK HKKAALLLDHPNGDGLNAIHLAMMSNSLPCLLLLVAAGADVNAQEQKSGRTALHLAVEHDN ISLAGCLLLEGDAHVDSTTYDGTTPLHIAAGRGSTRLAALLKAAGADPLVENFEPLYDLDDSWE NAGEDEGVVPGTTPLDMATSWQVFDILNGKPYEPEFTSDDLLAQGDMKQLAEDVKLQLYKLLE IPDPDKNWATLAQKLGLGILNNAFRLSPAPSKTLMDNYEVSGGTVRELVEALRQMGYTEAIEVI QAASSPVKTTSQAHSLPLSPASTRQQIDELRDSDSVCDSGVETSFRKLSFTESLTSGASLLTLNKMP HDYGQEGPLEGKI”
